# Secrets of Homœopathy—Hahnemann and His System

**Published:** 1845-10

**Authors:** 

**Affiliations:** Dramburg


*The Secrets of Homœopathy—Hahnemann and his System.* By Dr. SCHUBERT, of Dramburg. We hear it continually asserted that Hahnemann placed no confidence in the powers of nature in curing disease; but from my intercourse with him, I am quite satisfied that no physician ever trusted more to the *vis medicatrix naturæ.* It requires, indeed, but very little reflection to enable us to perceive that it was through the closest acquaintance with the curative powers of nature that Hahnemann was led to adopt his new system of medicine. I have heard him declare that he looked with contempt upon medical practice, and he thought that a patient would be none the worse if left to himself. He had a thorough conviction that all curable diseases might, under proper attention to diet, be removed by the efforts of nature alone; he looked upon these as his sheet-anchor. On one occasion he said to me—"I give medicines but very seldom, although I always prescribe small powders! I do this for the sake of keeping up in the patient's mind the firm belief that each powder contains a particular dose of some medicine! Most patients will get well by adopting a simple mode of living, and by placing a boundless confidence in their medical attendants. Ordinary practitioners know nothing of this practically, although they are always talking of the healing powers of nature. If a patient recover under their treatment, they immediately ascribe it to the nauseous drugs which they have poured into him, although these commonly do more harm than good." He never hesitated to promise recovery to every patient without concerning himself about the nature of the malady; and I have seen some most ludicrous results follow these predictions. His plan was to demand for the cure in the shape of a fee, a good round sum—one-half to be paid down—*unlimited confidence* in his treatment—doses of sugar of milk, and a particular diet! The dieting, which simply consisted in the denial of all stimuli, he considered to be absolutely necessary in order to allow nature to have free play. *Unlimited confidence* in the treatment was his great support in carrying out this system; and he invariably insisted upon this from every patient, well knowing that it was the important secret of life and death in such cases. Further, he used to observe—"We must not attend patients for nothing, or let them have even a pennyworth of medicine gratuitously; the greater the sum paid for physic and physician, the greater is the confidence placed in both."—*London Med. Gaz.*, from *Casper's Wöchenschrift.—Ibid.*

